# The coupling between localized surface plasmons and excitons via Purcell effect

**DOI:** 10.1186/1556-276X-7-669

**Published:** 2012-12-07

**Authors:** Feng Wang, Dongsheng Li, Deren Yang, Duanlin Que

**Affiliations:** 1 State Key Laboratory of Silicon Materials and Department of Materials Science and Engineering, Zhejiang University, Hangzhou, 310027, People’s Republic of China

**Keywords:** Localized surface plasmons, Silicon-rich silicon nitride, Silver nanostructures, Average position of excitons, Luminescence

## Abstract

The coupling between localized surface plasmons (LSPs) within silver nanostructures and excitons in a silicon-rich silicon nitride (SiN_*x*_) matrix has been demonstrated via the Purcell effect. A simple model is employed for the estimation of the Purcell factor as well as the average position of excitons within a luminescence matrix. The estimated average position of the excitons is located at approximately 40 nm beneath the top surface of the SiN_*x*_ films. The approaches for further improving the optoelectrical properties of the luminescence matrix are anticipated based on the model we adopted. The optimization of the thickness of the luminescence matrix as well as the size and shape of metal nanostructures may be the alternative approaches. Besides, the application of multilayers with the luminescence matrix inserted between barrier layers (we defined it as confined structures here) may be also an available choice. Our work may provide a deep comprehension on the coupling between LSPs and excitons, which is not limited to a certain luminescence material but with unconfined structures.

## Background

As an effective approach to overcome the diffraction limit of classical optics due to the mismatch of energy and momentum between electrons and photons, localized surface plasmons (LSPs) referring to the collective electronic oscillations within the metallic nanostructures excited by the external radiation have captured much research interest [1-8]. The confinement of electromagnetic field at the subwavelength volume as well as the Purcell enhancement of this field on the order of the quality factor (*Q*) of the LSP resonance is achieved by the adoption of these polariton modes [9,10]. This Purcell enhancement effect is similar to the Purcell effect in a microcavity with a quality factor *Q*, where the coupling between an exciton and a microcavity mode is allowed by the field confinement within an ultrasmall volume *V*[10,11]. This effect can enhance the density of photon states, which is proportional to the spontaneous emission decay rate, leading to the Purcell factor (*F*_p_) enhancement of luminescence [10,12]. Obviously, this *F*_p_ can characterize the coupling efficiency indirectly, which is influenced especially by the sizes and species of metallic nanostructures, the energy of emitted photons (hν_D_ = hc/*λ*_D_), and the distance (*d*) between LSPs and excitons in a luminescence matrix [13]. The effects of these parameters on *F*_p_ as well as the estimation of the average position of excitons within the luminescence matrix are particularly important for the further optimization of the luminescence properties of the active matrix.

In this letter, an amorphous silicon-rich silicon nitride (SiN_*x*_) film, a promising candidate material of silicon-based light sources due to its superior photo-physical properties [12,14-18], is employed as the luminescence matrix investigated here. Silver (Ag) nanostructures are used for the demonstration of the coupling between LSPs and excitons in SiN_*x*_ due to their lowest absorption losses and a superior enhancement of the local electromagnetic field among all the metals with plasmon resonances at visible frequency, which is near the luminescence wavelength of SiN_*x*_ films. Both the relationships between *F*_p_ and *λ*_D_ and the estimation of the average position (*d*) of the excitons within SiN_*x*_ are provided based on a simple model.

## Methods

SiN_*x*_ films with a thickness of approximately 50 nm were deposited using a plasma-enhanced chemical vapor deposition technique onto the substrates of p-type Si (100) or quartz for various measurements. The preparation of SiN_*x*_ films has been described in detail in our previous paper [12]. After the deposition of SiN_*x*_, a Ag layer was deposited by magnetron sputtering, with the thickness regulated by the sputtering time (20, 40, 60, and 80 s). To form the Ag nanostructures with various dimensions and surface morphologies, a rapid thermal annealing (RTA) in argon at 500°C for 60 s was employed subsequently. We label the samples by the sputtering time of the Ag layer, e.g., Ag40 refers to the sample with the sputtering time of the Ag layer of 40 s. A SiN_*x*_ film without Ag was also fabricated as the reference sample (Ag0).

The ellipsometric parameters *ψ* and *Δ* can be obtained from the ellipsometric measurement (M-2000D, J. A. Woollam Co. Inc., Lincoln, NE, USA), from which the complex index of refraction (*n*) can be calculated using the equation of $n=\frac{\left[\sqrt{1-4\sin^{2}\left(\theta_{0}\right)\tan\left(\psi \right)e^{\mathrm{j\Delta}}+2\tan\left(\psi \right)e^{\mathrm{j\Delta}}+\tan^{2}\left(\psi \right)e^{\mathrm{j\Delta}}}\right]n_{0}\sin\left(\theta_{0}\right)}{\cos\left(\theta_{0}\right)\left[1+\tan\left(\psi \right)e^{\mathrm{j\Delta}}\right]}$, where *θ*_0_ and *n*_0_ stand for the angle of incidence and the complex refractive index of the ambient, respectively [19]. Meanwhile, the film thickness (*t*) can be obtained from the equation of $t=\frac{j\ln\left(e^{-j2\beta }\right)\lambda }{4\pi n\cos\left(\theta_{1}\right)}$, where $\beta =2\pi \frac{t}{\lambda }\sqrt{n^{2}-n_{0}^{2}\sin^{2}\left(\theta_{0}\right)}$ and *θ*_1_ represents the angle of refraction [19]. All these calculations, including the determination of the extinction coefficient (*k*) as a function of wavelength, have been integrated into the ellipsometric measurement system. Consequently, we can acquire the data of *t*, *n*, and *k* directly from ellipsometry by scanning over the angle (*θ*_0_) range from 65° to 75° in steps of 5° with the spectral (*λ*) range from 400 to 800 nm. The extinction spectra of the samples with and without Ag nanostructures were obtained using a Hitachi U-4100 spectrophotometer (Hitachi, Ltd., Chiyoda, Tokyo, Japan). The photoluminescence (PL) spectra of all the samples were excited by a 325-nm He-Cd laser using an Acton SpectraPro-2500i monochrometer (Acton Research Corporation, Acton, MA, USA).

## Results and discussion

**Figure 1:**
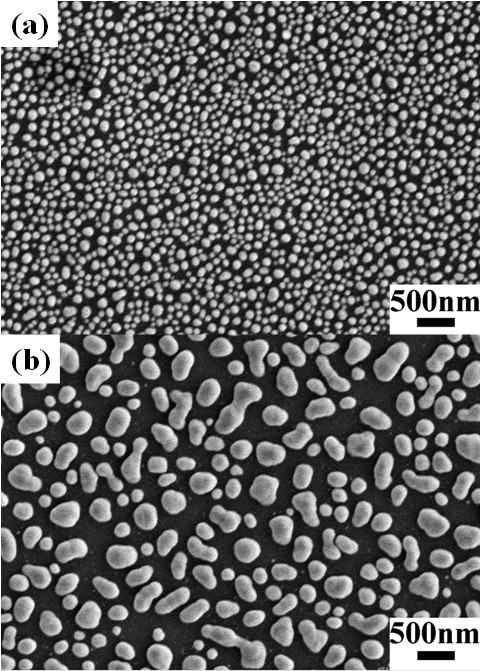
**SEM images of Ag nanostructures with different sputtering times.** (**a**) 40 and (**b**) 80 s. Discrete Ag nanostructures can be obtained after the RTA process.

**Figure 2:**
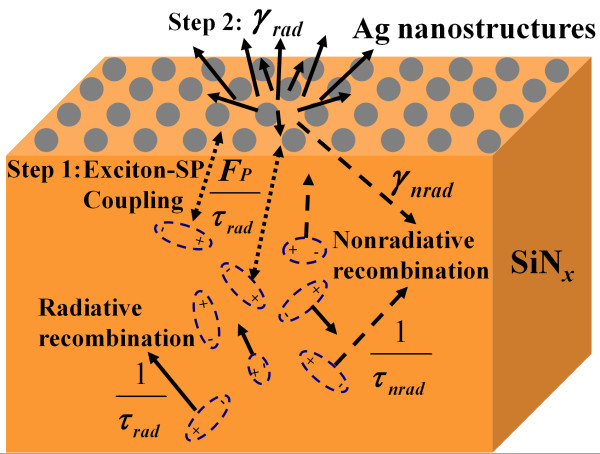
**Schematic diagram of the electron–hole recombination and coupling mechanism between LSPs and excitons in SiN**_***x***_**.** The interaction between LSPs and excitons can be treated as a two-step process: (1) the excitons transfer their energies to the LSP modes; (2) the energy from the LSP mode out-couples to radiated photons.

Figure 1 shows the scanning electron microscopy (SEM; Hitachi S-4800, Hitachi, Ltd.) images of Ag nanostructures with sputtering times of 40 and 80 s after the treatment of RTA. Dense Ag island films were obtained before the RTA process, where the sizes of Ag nanostructures were a little smaller than those with the RTA procedure. From the incomplete statistics, we obtained the radii (*r*_0_) of Ag40 and Ag80 as approximately 45 nm and approximately 110 nm, respectively. The larger size of the sample Ag80 might originate from the larger original size of the as-deposited Ag island film for Ag80. After the Ostwald ripening process [20], larger Ag particles could be obtained for the sample with a sputtering time of 80s than that with a sputtering of 40s. As have been investigated, the addition of Ag nanostructures can improve the luminescence efficiency of the active matrix [12]. It might result from the significant enhancement of the local electromagnetic field induced by the collective oscillation of excited free electrons surrounding the Ag nanostructures [11-13]. This enhanced local electromagnetic field will provide an additional path of recombination due to the high mode density in the LSP, from which the spontaneous emission rate can be increased significantly via the Purcell effect [11,12]. A possible mechanism of the LSP-exciton coupling is shown in Figure 2.

### Coupling between localized surface plasmons and excitons via Purcell effect

For the sample without the coverage of Ag nanostructures, the excitons generated in SiN_*x*_ films by optical or electrical pumping are terminated by radiative or nonradiative recombination with the internal quantum efficiency (*η*_int_) determined by the ratio of the radiative recombination rates (*κ*_rad_) to the nonradiative recombination rates (*κ*_nrad_) as *η*_int_ = *κ*_rad_ / (*κ*_rad_ + *κ*_nrad_) = *τ*_rad_^−1^/(*τ*_rad_^−1^ + *τ*_nrad_^−1^). After the addition of Ag nanostructures, the interaction between LSPs and excitons can be treated as a two-step process (shown in Figure 2). When the energy (ћ*ω*_ex_) of excitons is close to the electron vibration energy (ћ*ω*_SP_) of LSPs, the excitons transfer their energies to the LSP modes with the radiative recombination rates (*κ*_rad_) enhanced by the Purcell factor *F*_p_[4,11]. This process will compete with the nonradiative decay and enhance the PL decay rate (*κ*_PL_) significantly due to the large electromagnetic fields introduced by the high mode density in LSPs [21]. For the second step, the energy from the LSP mode will be out-coupled to radiated photons with the rate *γ*_rad_, as shown in Figure 2. This coupling will compete with the nonradiative loss (*γ*_nrad_) due to the absorption of Ag nanostructures, where the coupling efficiency can be defined as *η*_c_ = *γ*_rad_ / (*γ*_rad_ + *γ*_nrad_) [22]. This efficiency (*η*_c_) can be optimized via the modulation of the parameters of metal nanostructures, such as the size, shape, density, and the distance between metal nanostructures [22].

As has been mentioned above, the Purcell factor *F*_p_ can be used to characterize the efficiency of coupling into the SP mode [11,12,22] estimated as the ratio of the effective density of the SP modes *ρ*_SP_ to that of the radiation components *ρ*_rad_[23]. For the dipole placed at the distance *d* from the particle bottom surface and oriented in the *z* direction normal to the surface, the value of *ρ*_SP_ is given by [22,23]

(1) $$\rho_{\mathrm{SP}}=\frac{L\left(\omega \right)}{V_{\mathrm{eff}}}{\left(\frac{r_{0}}{r_{0}+d}\right)}^{6}$$

where the normalized line shape of the dipole oscillation *L*(*ω*) is

(2) Lω=ImεMω+2εD−1∫ImεMω+2εD−1dω

with *ε*_M_ and *ε*_D_ being the dielectric constant of the metal and surrounding media, respectively, which can be acquired from the ellipsometric data. A simple model is used for the estimation of the effective volume *V*_eff_ of SP modes, where the shape of the Ag nanostructures is a sphere in approximate with the radii (*r*_0_) equal to 45 and 110 nm for Ag40 and Ag80, respectively. The values of *V*_eff_ can be obtained via the following equation [22]:

(3) $$\begin{array}{l} V_{\mathrm{eff}}=\frac{{\int \int \int }_{r<r_{0}}\frac{1}{2}\varepsilon_{0}\frac{\partial \left(\omega \varepsilon_{M}\right)}{\partial \omega }E_{\mathrm{in}}^{2}d^{3}r+{\int \int \int }_{r>r_{0}}\frac{1}{2}\varepsilon_{0}\varepsilon_{D}E_{\mathrm{out}}^{2}d^{3}r}{\frac{1}{2}\varepsilon_{0}\varepsilon_{D}E_{\max}^{2}}\\ \;=\frac{4}{3}\pi r_{0}^{3}\left(1+\frac{1}{2\varepsilon_{0}}\right) \end{array}$$

where the dipole field inside (*E*_in_) and outside (*E*_out_) of the metal sphere with radius *r*_0_ can be obtained by solving the Laplace equation with proper boundary conditions at *r* = *r*_0_[24]. The maximum dipole field (*E*_max_) occurs at the surface of the metal nanostructures for this model. The value of *ρ*_rad_ is described as follows [22]:

(4) $$\rho_{\mathrm{rad}}=\frac{1}{3\pi^{2}}\left(\frac{2\pi }{\lambda_{D}}\right)\frac{1}{\omega }$$

with *λ*_D_ being the emission wavelength in the SiN_*x*_ matrix. Combining Equations 1 to 4, the Purcell factor can be obtained by

(5) $$F_{p}\left(\omega \right)=\frac{\rho_{\mathrm{SP}}}{\rho_{\mathrm{rad}}}=\frac{L\left(\omega \right)}{V_{\mathrm{eff}}}{\left(\frac{r_{0}}{r_{0}+d}\right)}^{6}{\left[\frac{1}{3\pi^{2}}{\left(\frac{2\pi }{\lambda_{D}}\right)}^{3}\frac{1}{\omega }\right]}^{-1}$$

**Figure 3:**
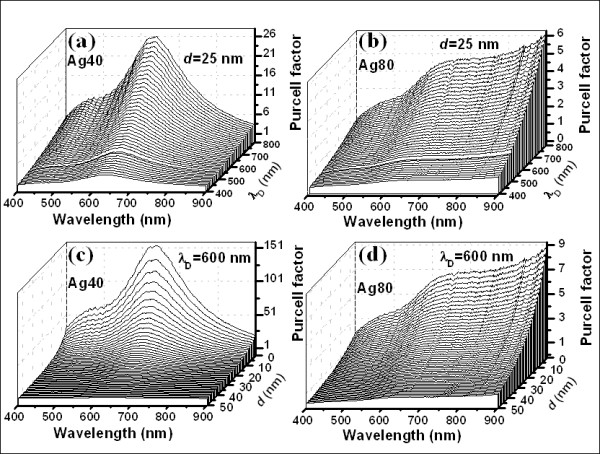
**The Purcell factors with different emission wavelengths and different LSP-exciton coupling distances.** (**a**) and (**c**) stand for Ag40, and (**b**) and (**d**) stand for Ag80. Both the distance between the Ag nanostructures and the excitons in SiN_*x*_ and the emission wavelength of the SiN_*x*_ matrix will influence the values of the Purcell factor.

Besides the parameters of the Ag nanostructures which will alter the values of *ρ*_SP_, both the distance (*d*) between the metal nanostructures and the excitons in SiN_*x*_ and the emission wavelength (*λ*_D_) of the luminescence matrix will influence the values of *F*_p_. Therefore, the influences of *λ*_D_ and *d* accompanied with the size (*r*_0_) of the Ag nanostructures on the values of *F*_p_ are simulated, as shown in Figure 3. By fixing the value of *d*, the influence of *λ*_D_ on *F*_p_ for Ag40 is compared with that for Ag80, as shown in Figure 3a,b. Obviously, the values of *F*_p_ are increased with the emission wavelength following the relationship of *F*_p_ ∝ *λ*_D_^3^ in both the cases. It means that the Purcell enhancement may be more conspicuous for the luminescence matrix with longer emission wavelength. A larger value of *F*_p_ for Ag40 is obtained compared to that for Ag80, which may result from the predomination of the volume term *r*_0_^3^ in Equation 5 over the factor ${\left(\frac{r_{0}}{r_{0}+d}\right)}^{6}$ here. It is clear that more effective LSP-exciton coupling can be achieved for Ag40 compared to Ag80. The influences of *d* on *F*_p_ are also checked in both Ag40 and Ag80 by setting the emission wavelength at 600 nm, as shown in Figure 3c,d. The values of *F*_p_ are increased with the decrease of *d*, and this increase is more obvious in Ag40 than that in Ag80. The influence of *d* may be much more significant than that of *λ*_D_ on *F*_p_ for the SiN_*x*_ matrix investigated here.

### Average position of excitons in SiN_*x*_ matrix

**Figure 4:**
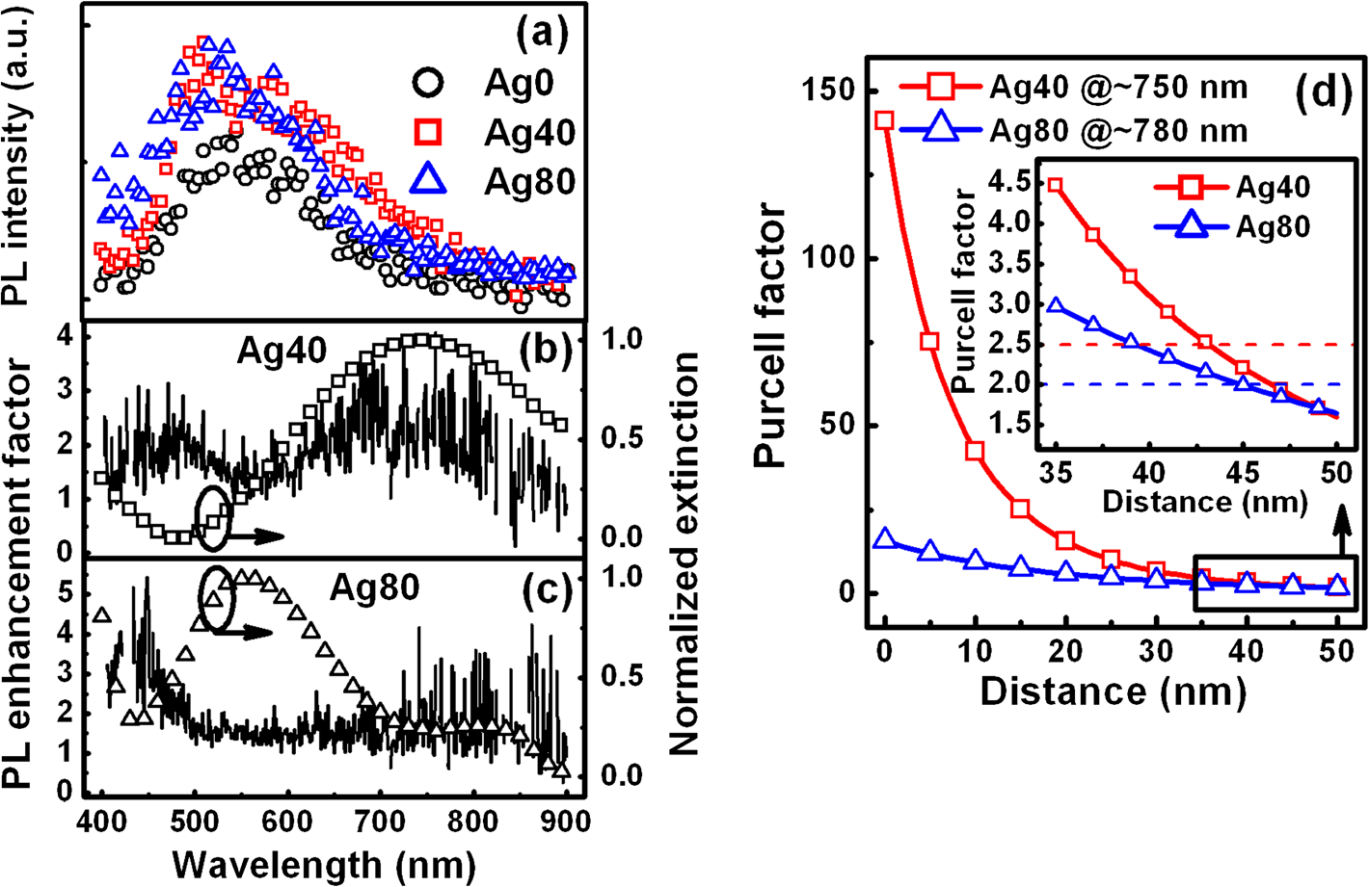
**The LSP resonance effects and the determination of the average position of excitons in SiN**_***x***_**.** (**a**) PL spectra for the samples with and without Ag nanostructures. PL enhancement factor (left axis) accompanied with the extinction spectra (right axis) for (**b**) Ag40 and (**c**) Ag80. (**d**) The Purcell factors vs. distance for Ag40 at *λ*_D_ = 750 nm and Ag80 at *λ*_D_ = 780 nm. The average position of excitons is located at 43 to 45 nm beneath the top surface of the SiN_*x*_ films.

From the discussions above, we can estimate the average position of excitons in the luminescence matrix with unconfined structures (the confined structure referred to the one with the active matrix inserted between two barrier layers) and optimize their luminescence properties conveniently based on this model. These matrixes may be SiO_*x*_, SiN_*x*_, Si nanocrystals within SiO_*x*_ or SiN_*x*_, ZnO, (In)GaN, and so on. For the estimation of the average position of excitons in our SiN_*x*_ matrix, PL spectra are measured for the determination of the value of *λ*_D_, as shown in Figure 4a. Broadband emission range from approximately 400 nm to approximately 850 nm with the central wavelength of approximately 600 nm could be observed. By dividing the PL intensity of Ag40 or Ag80 to that of Ag0, the PL enhancement factor (*F*_PL_) can be obtained. As can be seen in Figure 4b,c (left axis), two obvious peaks of the *F*_PL_ can be resolved, where the peak with shorter wavelength is induced by the improvement of light extraction from the active layer due to the minimum absorption of the Ag nanostructures. The peak with longer wavelength results from the coupling between LSPs and excitons in SiN_*x*_ confirmed by the extinction spectra, shown in Figure 4b,c (right axis), due to the consistence between this peak and the one of the dipole resonance, which is enhanced by the Purcell factor *F*_p_[11]. Obviously, the dipolar resonance peak of the Ag nanostructures redshifts gradually with the increase of their sizes. Correspondingly, the *F*_PL_ is located at approximately 750 nm with the value of approximately 2.5 for Ag40 and at approximately 780 nm with the value of approximately 2.0 for Ag80.

To find out the relationship between *F*_p_ and *F*_PL_, the originations of these two factors are considered. Both of them mainly result from the enhancement of the spontaneous emission rate, where the value of *F*_p_ can be rewritten as *F*_p_(*ω*) = *κ*_PL_^*^(*ω*)/*κ*_PL_(*ω*), with *κ*_PL_(*ω*) and *κ*_PL_^*^(*ω*) standing for the original and enhanced PL decay rates, respectively [25] . Consequently, the approximation relation between *F*_p_ and *F*_PL_ (*F*_P_ ≈ *F*_PL_) can be employed for the estimation of the average position of excitons (*d*) in SiN_*x*_ by plotting the curves of *F*_p_*vs*. *d* at the wavelength where the enhancement of dipole resonance occurred, as shown in Figure 4d. The average position of excitons is located at 43 to 45 nm beneath the top surface of the SiN_*x*_ films. Consequently, the optimized luminescence properties can be achieved via the optimization of parameters in the Equation 5 by the modulation of the sizes and shape of the Ag nanostructures. Both the optimal PL and electroluminescence efficiency of a SiN_*x*_-based light-emitting device are achieved by the addition of Ag nanostructures with the radii of approximately 50 nm from our experimental results (not shown here). Further attentions can be paid to the decrease of the distance between LSPs and excitons, which may be achieved via the optimization of the thickness of the luminescence matrix as well as the appropriate design of the luminescence structure. Multilayers with the active matrix inserted between barrier layers may be also an available choice.

## Conclusions

The coupling between LSPs and excitons in SiN_*x*_ has been demonstrated via the Purcell effect. When the energy of the excitons is close to the electron vibration energy of LSPs, the excitons can transfer their energy to the LSPs, in which the radiative recombination rates are enhanced by the Purcell factor. The relationships between the Purcell factors and the deposition parameters are illustrated, including the size of the Ag nanostructures, the energy of the emitted photons, and the distance between the LSPs and excitons in the SiN_*x*_ matrix. Further improvement on the luminescence efficiency can be achieved by decreasing the distance between LSPs and excitons and/or modulating the parameters of the metal nanostructures.

## Abbreviations

Ag: silver; *F*_PL_: PL enhancement factor; *F*_p_: Purcell factor; LSPs: localized surface plasmons; PL: photoluminescence; RTA: rapid thermal annealing; SEM: scanning electron microscopy; SiN_*x*_: silicon-rich silicon nitride; SiO_*x*_: silicon oxide.

## Competing interests

The authors declare that they have no competing interests.

## Authors’ contributions

FW performed the experiments, collected and analyzed the data, and wrote the paper. DL conceived the experiments, analyzed the results, and wrote the paper. DY and DQ helped with the data analysis and wrote the paper. All authors read and approved the final manuscript.
